# Prevalence and status of Lipoprotein (a) among Lebanese school children

**DOI:** 10.1038/s41598-020-77689-5

**Published:** 2020-11-26

**Authors:** Marie-Hélène Gannagé-Yared, Christina Lahoud, Nada Younes, Rima Chedid, Ghassan Sleilaty

**Affiliations:** 1 Department of Endocrinology, Faculty of Medicine, Saint-Joseph University, Beirut, Lebanon; 2 Department of Biostatistics and Clinical Research Center, Faculty of Medicine, Saint-Joseph University, Beirut, Lebanon; 3 Division of Endocrinology, Hotel-Dieu de France Hospital, Beirut, Lebanon

**Keywords:** Cardiology, Endocrinology

## Abstract

Lipoprotein a (Lp(a) is an independent risk factor for atherosclerotic cardiovascular disease. The prevalence of high Lipoprotein (a) (Lp(a)) in the Lebanese pediatric population is unknown. Our study aims to assess this prevalence and to study the relationship of Lp(a) with the lipid profile, age, body mass index (BMI) and socio-economic status (SES) in Lebanese schoolchildren. A total of 961 children aged 8–18 years (497 boys and 464 girls) were recruited from ten private and public schools in 2013–2014 using a stratified random sample. Schools were selected from the Greater Beirut and Mount Lebanon areas, and were categorized into three subgroups according to the schools’ SES status (high, medium, low). Lp(a) was assayed in 2018 on samples previously frozen at − 80 °C. Abnormal Lp(a) levels (≥ 75 nmol/L) were observed in 14.4% of the overall sample (13.5% for boys,15.3% of girls p = 0.56). The median of Lp(a) was 20(10–50) in the whole sample with no significant gender difference. No significant relationship was found between Lp(a) and age. However, Lp(a) was significantly correlated with BMI in whole sample, as well as in boys and girls (p = 0.02, p = 0.03, p = 0.03, respectively). A significant correlation was found between Lp(a) and non-HDL-C in the whole sample as well as in boys and girls (respectively p < 0.001,p = 0.024 and p = 0.03), but not with triglycerides and HDL-C. In a multivariate linear regression analysis, Lp(a) was only independently associated with BMI and non-HDL-C in boys and girls. Lp(a) was independently associated with BMI and non-HDL-C while no significant relationship was observed with age and sex confirming the strong genetic determination of Lp(a).

## Introduction

Dyslipidemia in children contributes to atherosclerosis and therefore can lead to cardiovascular disease (CVD) in adults at an early age. The recently published guidelines for the reduction of cardiovascular risk in children and adolescents^1^ recommend a universal screening for non-high-density lipoprotein cholesterol (non-HDL-C) in children aged between 9 and 11, followed by a second one between 17 and 21 The purpose of this screening is to identify and control dyslipidemia during childhood in order to reduce the CVD that begins at a young age^2^.

Other lipoproteins, mainly lipoprotein (a) (Lp(a)) are also identified as highly atherogenic and can subsequently contribute to atherosclerosis^3–7^. Lp(a) is a low-density Lipoprotein (LDL)-like particle in which apo B is covalently bound by a single disulfide bond to a glycoprotein apolipoprotein(a) (apo(a))^5^. Placed under potent genetic control^3,8^, its plasma concentrations vary considerably between individuals from different ethnicity with 2- to 3-fold higher levels in black population compared to white population^3,9^. Several recent studies report a causal role of Lp(a) in atherosclerotic CVD and valvular aortic stenosis^5,8,9^. In addition, due to the structural homology of one of its apo(a) domains with plasminogen, Lp(a) interferes with thrombolysis^10^. Therefore, its elevation plays an important role in the development of thromboembolic diseases^11^. Several meta-analyses concluded that high Lp(a) is an additional risk for incident stroke in adults^8,12^. Data suggest that high Lp(a) in youth also increases the risk of future atherosclerotic CVD, ischemic stroke, and possibly venous thromboembolic events^10,13,14^. All these effects are markedly increased in the presence of high low-density lipoprotein cholesterol (LDL-C) or low high-density lipoprotein cholesterol (HDL-C)^15^.

The 2011 panel on the reduction of cardiovascular risk in children and adolescents did not recommend routine Lp(a) screening, but rather suggested measuring Lp(a) in young people with a history of ischemic or hemorrhagic stroke, and those with a parental history of CVD not explained by classic risk factors^1^.

The data that support screening for Lp(a) is currently emerging^7^. However, to date, there is no established therapeutic strategy in lowering Lp(a) in youth.

Lp(a) is placed under potent genetic control^3,16^. Its plasma concentrations vary considerably between individuals from different ethnicity with 2- to 3-fold higher levels in black population compared to white population^3,17^. In addition, Lp(a) levels are relatively constant throughout life and are not influenced by age, gender, diet or lifestyle^8,18^.

Lp(a) has been poorly studied in Middle Eastern countries. A study published in 1991 showed higher Lp(a) levels in families with heterozygous familial hypercholesterolemia^19^. In Lebanon, 20% of adult volunteers recruited from hospital staff showed Lp(a) values greater than 30 mg/dL^20^, a threshold considered to be abnormal^21^. In another Lebanese study, Lp(a) was an independent predictor of coronary heart disease^22^. However, few studies have focused on Lp(a) in youth. The most comprehensive data in children come from the 3rd National Health Nutrition and Examination Survey (NHANES) which studied Lp(a) in 1505 subjects aged 4–19 years from different ethnic groups^17^. To date, Lp (a) levels in the pediatric population in Lebanon and the Middle East are still lacking.

The aim of this study is to estimate the prevalence of high Lp(a) in the Lebanese pediatric population and to study its relationship with the lipid profile [total cholesterol (TC), triglycerides and non-HDL-C], age, body mass index (BMI) and socio-economic status (SES).

## Materials and methods

### Population

This cross-sectional study is the continuum of a previous study that was carried on a sample of the Lebanese pediatric population^23^ and in which 969 school children were screened for a non fasting lipid profile (TC, triglycerides and HDL-C). In that study, 10 private and public schools were targeted for recruitment using a stratified random sample. The schools were selected in the Greater Beirut and Mount Lebanon regions, the two Lebanese areas that concentrate most of the Lebanese population. Recruitment and sampling were done between May 2013 and October 2014. Sampling was taken for 10 days (one whole day per school). Schools were classified as pertaining to a high, medium or low socioeconomic status (SES) based on annual tuition fees (for group 1 between $5000 and $7000; for group 2 between $3000 and $5000; and for group 3 between $1000 and $3000 or free, the last group corresponding to public or semi-public schools). Children suffering from any acute or chronic medical condition (more particularly diabetes or hypothyroidism), those on drugs which can affect the lipid profile (contraceptive pills, isotretinoin, oral corticosteroids, atypical antipsychotics or immunosuppressive therapies) as well as non-consenting children were excluded from the study.

On the day of the sampling, all the participants had a measurement of their height and weight done using the same calibrated device. BMI was calculated as weight in kilograms divided by height in meters squared (kg/m^2^). To account for variability by age and sex, all BMI measures were compared with age and sex-specific reference values ​​of the 2000 Centers for Disease Control and Prevention (CDC) growth charts in order to define weight status^24^. This comparison was chosen because of the lack of reference values in Lebanon. Normal or thinness BMI was defined as BMI for sex and age < 85th percentile, overweight as BMI for sex and age between 85 and 95th percentile, and obesity as BMI for sex and age ≥ 95th percentile^24^. The population was categorized into three age groups: 8–11 years, 12–14 years and 15–18 years.

The parents had already signed an informed consent to practice a lipid profile on their children. The project was approved by the ethics committee at Hôtel-Dieu de France university hospital, Beirut, Lebanon (CEHDF 1174). All methods were carried out in accordance with relevant guidelines and regulations.

The children's serum samples, collected in 2013–2014, have been stored since at – 80 °C, and were used for the Lp(a) analysis which was done in 2018. Eight subjects were excluded from our previous publication^23^ because of insufficient serum samples to perform the Lp(a) measurement.

### Biological parameters

#### Lipoprotein A

Lp(a) assay measurements were performed on samples previously frozen at − 80 °C. The assay is a particle-reinforced immunoturbidimetric assay (Cobas Integra 400 plus system, Roche Diagnostics). In this method, human Lp(a) agglutinates with latex particles coated with anti-Lp(a) antibodies. The precipitate is determined by turbidimetry at 659 nm. The measurement range varies from 7 to 240 nmol/L with a detection limit of 7 nmol/L. Samples > 240 nmol/L were diluted. According to the manufacturer, the threshold value which indicates an increased risk is 75 nmol/L. The conversion factor from nmol/L to mg/dL is nmol/L × 0.4167 = mg/dL.

#### Lipid profile

The measurement of TC, HDL-C and triglycerides was performed on a Vitros 5.1 FS machine (Ortho-Clinical Dignostics, Inc. Raritan, NJ, USA). Non-HDL-C was calculated by subtracting HDL-C from the TC.

#### Total testosterone

Total testosterone levels were measured only in boys using the Immulite 2000 automate (Siemens, USA). The sensitivity of the method is 20 ng/mL and the coefficient of variation < 10% for values > 150 ng/mL.

### Statistical analysis

The calculations for sample size were performed to calculate the prevalence of high Lp(a) taking into account the following criteria: an effect size-g-of 5%, accounting for the prevalence rate of high Lp(a) in different populations, a two-tailed alpha set to 5% and a 95% level of statistical power. The same calculation was applied in our previous publication performed on the same sample^25^.

Using the exact binomial distribution, 747 subjects are needed as the minimal sample size. The sample collected from different schools was 25% higher than the minimal required theoretical sample size, thus ensuring adequate statistical power.

Quantitative variables were tested for normality distribution using the Kolmogorov–Smirnov test. In case the variable departed significantly from normality, it was expressed as median and interquartile range (IQR) (quartile 1–quartile 3). Categorical and normally distributed continuous variables were expressed as frequencies and mean ± standard deviation, respectively. Original Lp(a) values reported semi-quantitavely as < 7 nmol/L were set to 6 nmol/L and were analyzed using non parametric methods. Chi-square test, independent *t* test, Mann–Whitney U test, Jonckheere–Terpstra and Mantel–Haenszel (linear by linear) tests were used in univariate analysis. Pearson correlation was used to assess the association between quantitative normally distributed variables, otherwise, Spearman’s correlation was used. 95% confidence intervals for correlation coefficients were derived by bootstrapping based on 10,000 samples.

A multiple linear regression model was built with natural logarithm (Ln) of Lp(a) as continuous dependent variable and age, Ln of BMI CDC percentile, Ln of lipid parameters, and SES status as independent factors. The model’s adjusted R^2^ and Cook’s influence statistics were calculated. The statistical analysis was performed using SPSS (IBM Corp. Released 2019, SPSS Statistics for Windows Version 26.0, Armonk, NY, USA).

## Results

### Anthropometric and socioeconomic characteristics of the population (Table 1)

**Table 1:** Baseline demographic, anthropometric and lipid characteristics of the total population, and boys and girls taken separately.

	Total population (n = 961)	Boys (n = 497)	Girls (n = 464)
Age	13.4 ± 2.9	13.4 ± 2.8	13.3 ± 3.0
8–11 years (n = 328)	34.1%	31.0%	37.5%
12–14 years (n = 339)	35.3%	39.0%	31.3%
15–18 years (n = 294)	30.6%	30.0%	31.3%
BMI (percentiles)	68.4 (37.1–89.1)	70.5 (39.7–91.8)	66.1 (35.1–84.6)
Obese (n = 121)	12.6%	15.9%	9.1%
Overweight (n = 171)	17.8%	19.9%	15.5%
Normal or thinness (n = 669)	69.6%	64.2%	75.4%
SES			
High (n = 251)	26.1%	27.4%	24.8%
Middle (n = 257)	26.7%	25.4%	28.2%
Low (n = 453)	47.1%	47.3%	47.0%
Lipid parameters			
TC (mmol/L)	4.00 (3.53–4.50)	3.95 (3.50–4.42)	4.10 (3.60–4.60)
Triglycerides (mmol/L)	1.08 (0.80–1.57)	1.07 (0.77–1.53)	1.08 (0.82–1.59)
HDL-C (mmol/L)	1.24 (1.10–1.50)	1.20 (1.04–1.44)	1.30 (1.10–1.50)
Non HDL-C (mmol/L)	2.70 (2.30–3.20)	2.60 (2.20–3.16)	2.74 (2.30–3.20)

Categorical variables are expressed as frequencies and percentages. Continuous variables with Gaussian distribution are expressed as mean ± standard deviation, or as Median with its interquartile range (1st quartile–3rd quartile) otherwise.

A total of 961 children, 497 boys and 464 girls (51.7% and 48.3% of the sample, respectively) were included in the study. The mean age was 13.4 ± 2.9 years with no significant difference between genders (p = 0.727). The median BMI percentile was 68.4 (37.1–89.1). The difference in BMI percentiles between boys and girls was significant [70.5(39.7–91.8) vs. 66.1(35.1–84.6) respectively, p = 0.012]. In the entire sample, 12.6% of the children were obese (15.9% of boys and 9.1% of girls) and 17.8% overweight (19.9% ​​of boys and 15.5% of girls), with a significant gender effect when comparing the 3 BMI categories (p = 0.001). Respectively, 26.1%, 26.7% and 47.1% of the children were coming from schools with high, medium and low SES without significant gender difference (p = 0.506). However, the proportion of obesity varied inversely with SES (5.2% in high SES schools, 11.7% in medium SES schools, and 17.2% in low SES schools, p < 0.0001). Finally, 261 girls (58.8%) had already had their menarche.

### Non-HDL-C, triglycerides, and HDL-C values in the entire sample and by gender

Results of non-HDL-C, triglycerides, and HDL-C in the entire sample and by gender are shown in Table 1. A significant difference is observed in the entire population for TC and HDL-C (p = 0.005 and p = 0.002, respectively).

In addition, in the age group 8–11, TG levels were higher and HDL-C levels were lower in girls compared to boys (p < 0.001 for both comparisons). At the opposite, in the age group 15–18, TG levels were higher and HDL-C levels were lower in boys compared to girls (p < 0.001 for both comparisons) (data not shown in table).

### Lp(a) values in the entire sample and by gender

Abnormal Lp(a) levels (≥ 75 nmol/L) were observed in 14.4% of the whole sample (13.5% in boys and 15.3% in girls, p value = 0.560) (Table 2). The proportions of children with a Lp (a) level ≤ 7 nmol/L and between 7 and 75 nmol/L were 21.2% and 64.4% respectively (20.7% of boys and 21.8% of girls had a Lp (a) level ≤ 7 nmol/L, and 65.8% and 62.9% respectively had values between 7 and 75 nmol/L) (Table 2).

**Table 2:** Categorization of Lp(a) in the total population and according to gender.

	Total population (n = 961) (%)	Boys (n = 497) (%)	Girls (n = 464) (%)	P value (gender)
**Total population**				
Lp(a) ≤ 7	21.2	20.7	21.8	0.56
7 < Lp(a) ≤ 75	64.5	66.0	62.9	
Lp(a) > 75	14.3	13.3	15.3	
**Age groups**				
8–11 years (n = 328)				
Lp(a) ≤ 7	23.2	20.8	25.3	0.53
7 < Lp(a) ≤ 75	67.1	70.1	64.4	
Lp(a) > 75	9.8	9.1	10.3	
12–14 years (n = 339)				
Lp(a) ≤ 7	19.5	20.1	18.6	0.87
7 < Lp(a) ≤ 75	63.7	63.9	63.4	
Lp(a) > 75	16.8	16.0	17.9	
15–18 years (n = 294)				
Lp(a) ≤ 7	21.1	21.5	20.7	0.57
7 < Lp(a) ≤ 75	62.6	64.4	60.7	
Lp(a) > 75	16.3	14.1	18.6	

Data are expressed as frequencies (percentages).

### Lp(a) values ​​ in relation with of age, BMI and SES categories

The values ​​of Lp(a) in the total population, and boys and girls, are shown in Table 3. The distribution of these values ​​according to age, BMI and SES categories are also shown in Table 3.

**Table 3:** Lipoprotein a (Lp(a)) in relation with age, socioeconomic status (SES) and Body Mass Index (BMI).

	Total population (n = 961)	Boys (n = 497)	Girls (n = 464)	p value (gender)
Total population	26 (10–50)	26 (10–48)	26 (9–55)	0.67
**Age groups**				
8–11 years (n = 328)	23 (10–43)	24 (10–42)	22 (7–44)	0.76
12–14 years (n = 339)	30 (10–55)	30 (10–52)	30 (10–62)	0.53
15–18 years (n = 294)	25 (10–55)	25 (10,49)	26 (9,61)	0.49
P value (age groups)	0.106	0.526	0.099	
**SES**				
High (n = 251)	24 (11–48)	25 (11–44)	22 (11–56)	0.51
Middle (n = 257)	24 (8–45)	23 (6–44)	25 (8–47)	0.76
Low (n = 453)	28 (10–57)	27 (11–56)	29 (9–57)	0.96
p value (SES groups)	0.132	0.131	0.565	
**BMI percentiles**				
Obese (n = 121)	29 (10–52)	26 (11–59)	30 (7–40)	0.72
Overweight (n = 171)	28 (11–56)	28 (13–50)	31 (9–57)	0.79
Normal or thinness (n = 669)	25 (9–49)	25 (8–46)	24 (9–55)	0.29
p value (BMI groups)	0.307	0.137	0.882	

Lipoprotein (a) (Lp(a)) levels (nmol/L) are expressed as Median with its interquartile range (1st quartile–3rd quartile). p values for the comparisons of lipid parameters between boys and girls are derived from the Mann–Whitney U test. p values for age groups, Body Mass Index (BMI) categories and socioeconomic status (SES) are derived from the Jonckheere–Terpstra test.

There is no significant difference in Lp(a) levels between boys and girls when analyzing the 3 age subgroups separately (Table 3). Similarly, there is no difference in Lp(a) values between boys and girls when the analysis is performed separately in both the 3 BMI and the 3 SES groups (Table 3).

### Lp(a) in relation with age, BMI and SES

There is no significant correlation between Lp(a) and age in the whole sample, and in boys and girls when analyzed separately [Spearman’s rho = 0.053 (− 0.011; 0.115), 0.045 (− 0.041; 0.130) and 0.062 (− 0.031 ; 0.155) respectively]. On the other hand, there is a significant correlation between Lp(a) and BMI in the whole sample as well as in boys and girls [Spearman’s rho = 0.098 (0.035; 0.161), 0.097 (0.010; 0.182) and 0.099 (0.007; 0.189), respectively]. Lp(a) is not correlated with SES (Spearman’s rho = 0.049 (− 0.014; 0.110).

### Lp(a) in relation with the other lipid parameters

The correlation of Lp(a) with triglycerides and with HDL-C is non-significant in the entire sample as well as in boys and girls [for triglycerides, Spearman’s rho = 0.040 (− 0.023; 0.103), 0.048 (− 0.043; 0.136), and 0.035 (− 0.057; 0.124); for HDL-C, Spearman’s rho = − 0.016 (− 0.079; 0.048), − 0.026 (− 0.113; 0.063) and − 0.006 (− 0.097 ; 0.085) respectively]. On the other hand, there is a significant correlation of Lp(a) with TC and non-HDL-C in the entire sample [Spearman’s rho = 0.111 (0.048; 0.174) and 0.122 (0.060; 0.181) respectively]. This significant correlation is also found in boys [Spearman’s rho = 0.088 (− 0.002; 0.178) and 0.101 (0.014; 0.187, respectively] and in girls [Spearman’s rho = 0.132 (0.042; 0.220) and 0.139 (0.050; 0.227), respectively].

### Lp(a) in relation to menarche and testosterone

The correlation between Lp(a) and testosterone in the 328 boys with a testosterone level above the detected threshold of > 20 ng/mL is not significant [Spearman’s rho = − 0.042 (− 0.151 ; 0.068)]. In addition, Lp(a) did not differ between girls who already had their menarche and those who did not [27 (9–57) vs. 22 (8–46) nmol/L, p = 0.178].

### Multiple linear regression analysis with Lp (a) as the dependent variable

Using multiple linear regression, Lp(a) was not independently associated with sex, age or SES. Only BMI and non HDL-C remained significantly associated with Lp(a) (Table 4).

**Table 4:** Multiple linear regression analysis with lipoprotein a (Lp(a)) as a dependent variable and gender, age, BMI, SES, non-HDL-C, triglycerides and HDL-C as independent variables.

	B	Std. error	p value
(Constant)	1.084	0.575	0.060
Ln(triglyceride)	-0.090	0.086	0.300
Ln(HDL-C)	0.164	0.163	0.315
Ln(non-HDL-C)	0.537	0.139	0.**000**
Ln(BMI)	0.432	0.204	**0.034**
SES	0.059	0.042	0.158
Age	0.007	0.013	0.618
Gender	0.021	0.067	0.751

Due to their positively skewed distribution, lipid parameters and BMI were entered the model using their natural logarithmic transform.

Model’s R^2^ = 0.02.

*BMI* Body Mass index, *SES* socio-economic status (1 = high, 2 = medium, 3 = low), gender (male = 1; female = 2), *B* unstandardized linear coefficient.

## Discussion

The current study assessed the prevalence of high Lp(a) levels in a representative sample of the Lebanese pediatric population and its relationship with age, BMI, SES and lipid profile (TC, triglycerides and non -HDL-C). High Lp(a) levels (≥ 75 nmol/L) were found in 14.4% of the sample, with no significant difference according to gender. Of note, the 75 nmol/L cut -off value, which is associated with an increased cardiovascular risk, was established in adult subjects only. In addition, most of the previous studies expressed Lp(a) values in mg/dL, and its conversion to nmol/L is inaccurate since measurement of Lp(a) mass concentration with the previously available commercial assays varied with the number of apo(a) kringle repeats, leading either to overestimation or underestimation of Lp(a)^26^. More Recent Lp(a) assays measure the particle concentration in nmol/L independently of the apo(a) component and are therefore more appropriate in predicting the cardiovascular risk^3^. In practice, extrapolation of Lp(a) values ​​from mg/dL to nmol/L is incorrect and the interpretation of the literature results is not obvious.

The 14.4% proportion of Lp(a) ≥ 75 nmol/L in the current sample is slightly lower compared to the 18.4% proportion published by the NHANES study in Caucasian children aged 4–16 years with no family history of CVD and with a Lp(a) > 30 mg/dL^17^. This observed small difference could be explained by several factors: use of different Lp(a) assays, absence of data on the parental history of CVD in the current study, or ethnic differences. In another cross-sectional study^27^ 257 children at high risk for CVD were referred to a preventive cardiology clinic. The authors found 42.8% prevalence of high Lp(a). This could be explained by the bias of the recruited population. Of note, 21% of the included subjects in the current series had Lp(a) levels ≤ 7 nmol/L, but the significance of this observation remains to be elucidated.

Lp(a) was not related to age in our population, a finding consistent with the NHANES study 17) in which Lp(a) levels > 30 mg/dL did not increase with age except in Mexican Americans. Similarly, in Asian Indian Descendants, no significant association was found between Lp(a) and age^28^. In contrast, in our previous report carried out on this same sample, the lipid profile (non-HDL-C, triglycerides, HDL-C) was influenced by age and sex^23^: non-HDL-C and triglycerides were higher in girls in the age group 8–11 years compared to the age group 15–18, while the opposite was observed in boys. These differences might be explained by a strong genetic determinant of Lp(a)^3^.

Lp(a) levels did not differ by sex neither in the whole series nor in age subgroups, corroborating both the NHANES results for Lp(a) rates before the age of 20^17^ and the results in the adult population in Copenhagen^29^.

Interestingly, the current study showed a significant relationship between Lp(a) and BMI, independently of age and SES effects. In our previous report^23^, non-HDL-C and triglycerides were also positively correlated with BMI in boys and girls, suggesting that obesity is harmful to all lipid parameters. Similarly to our study, the 3^rd^ NHANES study reported higher numbers of youth with elevated Lp(a) in the subgoups with a high BMI^17^. In another study, Glowinska et al.^30^ found that young obese, hypertensive and diabetic patients have a Lp(a) twice as higher than in control group. Conversely, in a Taiwanese study^31^ performed on 1283 school children aged 12–16, none of the anthropometric measures (body weight, waist and hip circumference) were significantly correlated with Lp(a) levels. Likewise, in another report, there was no significant association between Lp(a) and BMI in children of Asian Indian descendants and their Caucasian neighbors^28^. Finally, Brandstatter el al.^32^ observed that after a 3-week hypocaloric diet in children, a 6.6% decrease in body weight leads to ~ 20% decrease in Lp(a) levels, which is comparable to the decline seen in LDL-C and triglycerides. These findings suggest that BMI is strongly related to Lp(a) and that ethnicity may affect this relationship.

We found no difference in Lp(a) according to SES. The relation of SES with Lp(a) level has been poorly studied. In Indian descendants, the family daily income was negatively correlated with Lp(a) while it was positively correlated with Lp(a) in their Caucasian neighbors^28^. While ethnic differences could explain this discrepancy, it is also possible that the unavailability of individual family income in the current study could have confounded the effect of SES on Lp(a).

In the current series, we analyzed Lp(a) relationship with lipid parameters: it was positive for non-HDL-C and absent for triglycerides and HDL-C. In the same line, Meabe et al.^33^ found a positive correlation between Lp(a), LDL-C and apo B in a cohort of Spanish children. Similarly, in both the Taiwanese study^31^ and the one performed on Indian descendants^28^, LDL-C and ApoB levels were significantly positively associated with Lp(a) levels, supporting the fact that, irrespectively of ethnicity, high Lp(a) levels are associated with high LDL-C levels, and that LDL metabolism is involved in the synthesis of Lp(a). Surprisingly, Qayum et al.^27^ did not find a significant association between Lp (a) and LDL-C in a cohort of high risk cardiovascular children. This could be due to the smaller size of their cohort or to higher cardiovascular risk of their population. High Lp(a) levels could explain the therapeutic failure of statins since by inducing an apparent resistance to these drugs^34^. Excess Lp(a) can promote the initiation and early development of atherosclerotic plaque which can be accelerated by the presence of other pro-atherogenic factors, such as LDL-C; this leads to a synergistic interaction which increases the cardiovascular risk. Hence, in patients with high LDL-C, Lp(a) levels would be an important factor in determining the risk of CVD and the severity of its course. In another study, Sharma et al.^35^ found that in overweight/obese African American children and adolescents, Lp(a) is positively and independently associated with HDL-C but not with the other lipoproteins. The apparent discrepancy in Lp(a) plasma levels and its relationship with lipid profile could result from factors pertaining to each population, such as diet, genetic factors, and race.

The current study is cross-sectional by design. it may be interesting to follow-up the children included in this study longitudinally through adulthood to eventually assess the long-term clinical consequences of high Lp(a) levels during childhood and adolescence. The study also has some limitations. First, it is difficult to compare the observed results to those reported in the literature where Lp(a) is expressed in mg/dL for the reasons already discussed earlier. Reporting the results in nmol/L has recently been described as being much more consistent with the prediction of clinical outcomes. Second, there are no established reference values in children, which makes the interpretation of the results difficult. Finally, information on the family history (dyslipidemia, diabetes, cardiovascular diseases) could not be collected in our sample to account for it during the analysis.

In conclusion, the prevalence of high Lp(a) in our population was 14.4% and comparable to the results of non-Hispanic whites in the American NHANES study. Lp(a) levels were not influenced by gender nor by age, but were positively related to BMI and non-HDL-C. In the future, we suggest measuring Lp(a) in children and adolescents at high cardiovascular risk, while awaiting results from long-term studies investigating potential changes in Lp(a) between childhood and adulthood.

## Data Availability

The data described in the manuscript are available upon request with the corresponding author Marie-Hélène Gannagé-Yared (Department of Endocrinology, Saint-Joseph University, Beirut, Lebanon Email: mariehelene.yared@usj.edu.lb.
